# Supplementary material for: Power pose effects on approach and avoidance decisions in response to social threat
Source: PLoS One. 2023 Aug 18;18(8):e0286904. doi: 10.1371/journal.pone.0286904 (PMC10437884; doi:10.1371/journal.pone.0286904)
Supplement: S1 Appendix — Regarding the methods section, the S1 Appendix contains detailed information on how our task compares to other approach-avoidance tasks, the cover story, pose instructions, debriefing, self-report questionnaires and data analysis. In the results sections, we report self-report questionnaire values for both experimental groups, result tables for all generalized linear mixed effects models, results in terms of ANOVA and t-tests for proportion of choice, and all results for movement kinematics. These include descriptive statistics for movement kinematics, linear mixed effect model and ANOVA results for both reaction time and movement duration. (PDF) [file pone.0286904.s001.pdf]

Power pose effects on approach and avoidance decisions in response to social threat

S1 Appendix
Supplementary Information on Methods and Results

Hannah Metzler, Emma Vilarem, Adrian Petschen, Julie Grèzes

Regarding the methods section, the Appendix contains detailed information on how our task compares to other approach-avoidance tasks, the cover story, pose instructions, debriefing, self-report questionnaires and data analysis. In the results sections, we report self-report questionnaire values for both experimental groups, result tables for all generalized linear mixed effects models, results in terms of ANOVA and t-tests for proportion of choice, and all results for movement kinematics. These include descriptive statistics for movement kinematics, linear mixed effect model and ANOVA results for both reaction time and movement duration.

Methods

Approach-avoidance task

The approach-avoidance task we used [1] allows investigating spontaneous action decisions between two competing targets for action (two empty chairs) in the presence of two task-irrelevant individuals, one neutral and another one displaying a negative facial expression (either fear or anger). This experimental approach differs from existing compatibility paradigms (e.g. the Approach-Avoidance Task, AAT [2,3]) that require subjects to pull or push a joystick to categorize either the valence or some other task-irrelevant feature of stimuli, such as faces' gender [for an overview, see 4]. In contrast to our free choice paradigm, in the AAT, participants have to perform one explicitly instructed action, which either results in approaching positive/avoiding negative stimuli (compatible trials), or approaching negative/avoiding positive stimuli (incompatible trials). Reaction times are usually faster in compatible than in incompatible trials [2,3,5,6].

However, a number of factors were shown to affect how subjects respond to the same emotional stimulus, such as the subject's self-representation in space [7], or the explicit label ("approach"/"avoidance") assigned to the response movement [8,9]. For example, the same movement (e.g. arm flexion) has been labeled as either approach (e.g. for retrieving something desired) or avoidance (e.g. for moving away from a spider) across different studies [4]. Such factors could explain why AAT paradigms yielded contradicting findings regarding responses to angry and fearful expressions [6,10–13]. In contrast to these previous paradigms, the task by Vilarem et al. [1] allows participants to freely choose among alternatives in a scene representing an everyday environment, without the constraint of instructions, arbitrary movements or response labels. As in everyday life, it allows more closely examining how different alternatives for action compete to determine spontaneous approach-avoidance responses to emotional displays.

Procedure

Cover-story. At the beginning of the instructions, the male experimenter informed participants that they were going to participate in two separate studies, the first investigating spontaneous action choices and the second investigating effects of body postures on heart rate. Regarding this second study, participants were told that their heart rate needed to be registered for a total duration of 10 minutes while they held a certain pose. Participants were told that this total duration was divided in five separate 2-minute intervals, inserted between the blocks of the action choice task. According to our cover story, this served to avoid discomfort. To familiarize themselves with the relatively difficult action choice task, they would perform the first 5 blocks without pose. Thereafter, they would adopt a pose for 2 minutes before each of the last 5 blocks. Participants were further told that these 2 minutes breaks would help prevent tiredness of the eyes towards the end of the experiment from continuously focusing on the fixation cross.

Pose instructions. Pose instructions were only provided after participants had completed the first session (5 task blocks). After randomly determining the pose condition for each participant via a MATLAB function, the experimenter verbally provided instructions for either the expansive or contracted pose, taking care not to demonstrate it. Ensuring that the experimenter did not know the participant's pose condition until the last minute of the instructions was meant to minimize possible experimenter biases. The pose instructions for the expansive condition was: "Spread your legs to shoulder width and turn your feet outward. Place your hands on your hips with the thumb backwards and keep your elbows approximately parallel. Look straight ahead

and don't tilt your head downwards. The posture needs to be comfortable." For the contracted condition, it was: "Cross your legs and put one foot next to the other. Now, lay one arm across your belly and place the other one on top, but do not cross your arms. Look at the floor in front of you and relax your back and shoulders." After providing these instructions, the experimenter mentioned that he would observe the participants during posturing via a small camera to verify that it was correct and similar each time, and left the room before participants started the second session.

Debriefing. At the end of the experiment, participants were carefully debriefed regarding their decision strategies during the action choice task and their ideas about the purpose of both studies. Only 12 participants mentioned facial expressions as one of the factors influencing their decisions, but none of them specifically mentioned only anger and fear when directly asked which expressions they had recognized. Around half of participants listed a range of negative and positive emotions or simply noticed changes of eyebrows and the mouth.

Given that pose effects in previous studies may be explained by demand effects, it is important to assess if participants believed our cover-story, or had suspicions about the purpose of the study, for instance because they were familiar with power pose research. To assess such suspicions, we asked the following three debriefing questions:

1. Did you have any particular sensation or thought during the posturing task?
2. Did you think about objectives of the study we have not yet explained to you?
3. Did you perceive an influence of the posture task on the action task, or an influence in the reverse direction?

Only two participants correctly suspected a link between the action and the pose study in response to question 3. Excluding them from the analysis did not alter the results.

Self-report questionnaires

In order to control that participants from the expansive and contracted group did not differ significantly on personality trait or state measures, all participants completed the French version of the State-Trait Anxiety Inventory (STAI [14]), the Behavioural Inhibition and Activation System Scales [15], the Rosenberg Self-Esteem Scale [16], and four scales (PA, LM, HI, DE) from the Interpersonal Adjective List – Short Version [17] translated to French. For the latter, a dominance and affiliation score were calculated as (PA + reversed HI)/2 and (LM + reversed DE)/2, respectively. The state version of the STAI was filled out after signing consent forms at the beginning of the session, and all other questionnaires were filled out online prior to the testing day.

On the scales of the Interpersonal Adjective List, participants rated how the respective personality trait generally applied to them on a visual analogue scale with endings marked as "not at all" and "totally" ("pas du tout" and "tout à fait"). A native German speaker fluent in French translated the original German items to French, and verified that the translations had the intended meaning with two native French speakers. The original items and their translations for the four items per scale were: PA: direkt = direct, dominant = dominant, durchsetzungsfähig = affirmatif, selbstsicher = sûr de soi; LM: herzlich = chaleureux, einfühlsam = empathique, harmoniebedacht = conciliant, rücksichtsvoll = attentionné; DE: rücksichtslos = sans égard pour autrui, gefühlskalt = froid, gleichgültig = indifférent, unbarmherzig = sans pitié; HI: konfliktscheu = soumis, schüchtern = timide, still = calme, unauffällig = discret.

Data analysis

Random effects structure in linear mixed effects models. All models (on choice, initiation time and movement time) included a random intercept per participant to account for the within-

subject design, and if possible a random intercept per stimulus (i.e. pair of actors). Whereas the model for choice and movement duration converged with both random intercepts, the model for initiation time allowed only inclusion of a random intercept per participant. No higher random-effects structures (including uncorrelated intercepts and slopes, or only random slopes for the highest-order within-subject factor interaction of interest [18] converged without a singular fit, implying that the data did not support more complex random effect structures.

Movement kinematics. In addition to predictors in the generalized linear mixed-effects model (LMEM) on choice, the LMEMs on movement kinematics (initiation time and movement duration) included the within-subject predictor side (moving away vs. toward to the emotional actor, i.e. the dependent variable of the choice analysis). Akin to the predictor emotion, we used deviation coding (-0.5, 0.5) for side with toward as the baseline category. This implies that all estimates for side represent a main effect.

For both kinematic measures, we fitted two different LMEMs using restricted maximum likelihood (ML) estimation. We started by fitting a model on only emotional trials including the fixed effects emotion, side, session and pose and their interactions. As this first model revealed no main effects or interactions of emotion and side, we then grouped anger and fear together to compare threat vs. neutral trials. The second model was thus run on all trials including the fixed effects threat, session and pose and their interactions, with deviation coding for threat with neutral as the baseline category. To assess significance of fixed effects in the LMEMs for kinematic measures, we calculated t- and p-values using the Satterthwaite approximation for degrees of freedom implemented in the package lmerTest [19].

Results

Self-report questionnaires

Scores from participants in the expansive and contracted group did not differ significantly on any of the personality trait or state measures (all p-values>0.109. all effect sizes d< 0.36). Since the absence of a significant difference does not imply actual equivalence, we conducted equivalence tests [20] with d=-0.3 and d=0.3 as equivalence bounds for the smallest meaningful difference. None of the equivalence tests were significant (all p-values >0.130), i.e. the confidence intervals of all measures crossed one of the equivalence bounds, which implies that we could not assume statistical equivalence of the two groups on any of the measures. We thus included all measures as covariates into the model predicting choice as a function of emotion, session and pose. This did not change the pattern of results, i.e., previously significant effects remained the only significant effects.

Sensitivity analysis for proportion of choice

We conducted a sensitivity analysis to determine the minimal effect-size we could reliably detect with a power of 80% using the final sample size of n=79. This yielded a minimal effect size η^2_p=.10 (f=0.33 in G-Power), meaning we had at least 80% power for effects larger than this, and less than 80% for smaller effects. Investigating effects of emotion, session and pose on choice with an ANOVA yielded the same results as the generalized LMEM (see Table A and section “ANOVAs and post-hoc comparisons on proportion of choice”. Although LMEMs generally have higher statistical power, we chose to calculate power via ANOVA, because power calculation for LMEMs require simulations [21]. This sensitivity analysis thus likely underestimates the true statistical power of our analysis. The effect-size of the emotion x session x pose interaction in the ANOVA on choice ($F(1, 77) = 7.05$, p=.010, η^2_p = .084, η^2_G= .014) was slightly smaller than the minimal effect-size of η^2_p=.10 which we could detect with

80% power with our sample size. Assuming that the detected effect size corresponds to the true effect-size, this implies that we had a chance of approximately 75% to detect different behavioral changes in the two groups in response to the two emotions (such as the difference in avoidance of anger and fear getting larger in one group, but smaller in the other).

Generalized linear mixed-effects model proportion of choice

Table A. Parameter estimates and significance tests for model of choice away vs. toward.

Model of choice away vs. toward

**Expansive** pose **and session 1 as baseline**				
Predictors	*Odds Ratios*	*CI*	*z*	*p*
(Intercept)	1.06	1.01 – 1.12	2.57	0.010
Emotion	1.09	1.01 – 1.17	2.20	0.028
Session	1.00	0.94 – 1.05	-0.11	0.914
Pose	1.00	0.94 – 1.06	-0.07	0.944
Emotion x Session	0.96	0.86 – 1.07	-0.78	0.435
Emotion x Pose	0.99	0.88 – 1.10	-0.27	0.789
Session x Pose	1.03	0.96 – 1.12	0.87	0.386
Emotion x Session x Pose	1.19	1.02 – 1.38	2.18	0.029
**Contracted pose and session 1 as baseline**				
Predictors	*Odds Ratios*	*CI*	*z*	*p*
(Intercept)	1.06	1.01 – 1.12	2.44	0.015
Emotion	1.07	0.99 – 1.16	1.78	0.074
Session	1.03	0.98 – 1.09	1.11	0.267
Pose	1.00	0.94 – 1.07	0.07	0.944
Emotion x Session	1.14	1.02 – 1.27	2.30	0.022
Emotion x Pose	1.01	0.91 – 1.13	0.27	0.789
Session x Pose	0.97	0.89 – 1.04	-0.87	0.386
Emotion x Session x Pose	0.84	0.72 – 0.98	-2.18	0.029
**Expansive pose and session 2 as baseline**				
Predictors	*Odds Ratios*	*CI*	*z*	*p*
(Intercept)	1.06	1.01 – 1.11	2.43	0.015
Emotion	1.04	0.97 – 1.13	1.07	0.284
Session	1.00	0.95 – 1.06	0.11	0.914
Pose	1.03	0.97 – 1.10	0.98	0.327
Emotion x Session	1.04	0.94 – 1.16	0.78	0.435
Emotion x Pose	1.17	1.05 – 1.30	2.81	0.005
Session x Pose	0.97	0.89-1.04	-0.87	0.386
Emotion x Session x Pose	0.84	0.72-0.98	-2.18	0.029
**Contracted pose and session 2 as baseline**				
Predictors	*Odds Ratios*	*CI*	*z*	*p*
(Intercept)	1.10	1.04 – 1.15	3.68	<0.001
Emotion	1.22	1.13 – 1.32	5.02	<0.001
Session	0.97	0.92 – 1.02	-1.11	0.267
Pose	0.97	0.91 – 1.03	-0.98	0.327
Emotion x Session	0.88	0.79 – 0.98	-2.30	0.022
Emotion x Pose	0.86	0.77 – 0.95	-2.81	0.005
Session x Pose	1.03	0.96 – 1.12	0.87	0.386

Emotion x Session x Pose	1.19	1.02 – 1.38	2.18	0.029
**Random Effects**				
σ^2	3.29			
$\tau_{00\ subject}$	0.01			
$\tau_{00\ stim\_pair}$	0.00			
ICC	0.00			
$N_{subject}$	79			
N_{stim_pair}	10			
Observations	41780			
Marginal R^2 / Conditional R^2	0.001 / 0.003			

ANOVAs and post-hoc comparisons on proportion of choice

In agreement with the results of the mixed model, the ANOVA on proportion of choices away (p(away)) revealed an effect of emotion ($F(1,77)=19.63$, $p<0.001$, $\eta^2_p=0.203$, $\eta^2_G=0.060$), and a significant three-fold interaction emotion*session*pose ($F(1,77)= 7.05$, $p=0.010$, $\eta^2_p=0.084$, $\eta^2_G=0.014$), with no other significant effects. To break down this interaction term, we assessed the effect of session and emotion separately in each pose group. The ANOVAs per pose group showed no significant emotion*session interaction in the expansive group ($F(1,39)=1.47$, $p=0.233$, $\eta^2_p=0.04$, $\eta^2_G=0.006$), in contrast to a significant interaction in the contracted group ($F(1,38)=6.17$, $p=0.018$, $\eta^2_p=0.140$, $\eta^2_G=0.024$).

Two-tailed t-tests comparing the change from session 1 to 2 against zero in each group showed that this interaction was driven by an increased tendency to avoid anger after holding a contracted pose (see Figure 3b, $t(38)=2.40$, $p=0.022$, $d_z=0.38$). In contrast, contracted poses induced no significant change for fear ($t(38)=-0.94$, $p=0.349$, $d_z=-0.15$). In the expansive group, p(away) did not change significantly between sessions for neither anger ($t(39)=-0.82$, $p=0.416$, $d_z=-0.13$) nor fear ($t(39)=0.67$, $p=0.507$, $d_z=0.11$).

Comparing these changes in p(away) from session 1 to 2 between poses revealed a significantly larger change in the contracted pose for anger ($t(77)=2.28$, $p=0.026$, $d=0.51$), but no significantly different change between pose for fear $t(77)=-1.14$, $p=0.256$, $d=-0.26$). Finally, although there was no significant change for fear, the difference in responses to anger vs. fear became larger after a contracted pose (t-test on change anger vs. fear: $t(38)=2.48$, $p=0.017$, $d_z=0.40$), while it became smaller but not significantly so after an expansive pose ($t(39)=-1.21$, $p=0.23$, $d_z=-0.19$).

Starting from a non-significant proportion of away choices for anger in session 1 ($t(77)=0.25$, $p=0.804$, $d=0.056$), the increase in the contracted group resulted in a significant between-group difference in session 2 ($t(77)=-2.06$, $p=0.042$, $d=-0.46$). For fear, levels of avoidance differed significantly neither at baseline ($t(77)=-0.25$, $p=0.805$, $d=-0.06$), nor in session 2 $t(77)=1.50$, $p=0.138$, $d=0.34$).

In summary, starting from similar baseline levels in avoidance of anger and fear, the emotion effect only changed significantly in participants who adopted the contracted pose in session 2. This change was mainly driven by increased avoidance of anger.

Initiation time

The LMEM on log-transformed initiation time (time from appearance of scene to first click) *log(initiation time) ~ (1|subject) + emotion x side x session x pose* fit the data significantly better than the null-model ($\chi^2(15)=1820$, $p<0.001$, deviance (-2LL) reduced 19498 from to 17678). Importantly, it revealed no significant main effect or interaction of emotion and side (all p's >0.436), nor any interaction of pose or session with emotion or side (all p's >0.117, see Table B,

and D for the same analysis using ANOVA). This indicates, first, that neither emotion and side, nor their interaction influenced initiation times in the expansive group in session 1, and second, that these effects did not significantly differ between poses or sessions.

We therefore examined all other effects in the model *log(initiation time) ~ (1|subject) + threat x session x pose* including all trials, contrasting threat-related facial expressions (anger and fear) with neutral ones (see Table C, and D for the same analysis using ANOVA). Again, the model fit the data significantly better than the null-model ($\chi^2(7)=3484.6$, $p<0.001$, deviance (-2LL) reduced from 29442 to 25958). With the expansive group and session 1 as baseline levels for the treatment contrasts, this model revealed quicker initiation times in session 2 as compared to session 1 (β=-0.16, 95% CI=-0.17 – -0.15, t=-45.35, p<0.001), which might indicate a learning effect across sessions. Although the significant session by pose interaction (β=0.06, 95% CI=0.05 – 0.07, t=11.44, p<0.001) hinted that this session effect (i.e. acceleration of initiation time from session 1 to 2) was less pronounced in the contracted group, it was also highly significant (β=-0.10, 95% CI=-0.11 – -0.10, t=-28.5, p<0.001). Overall, initiation time reduced from 405 ± 144 ms in session 1 to 364 ± 134 ms in session 2 (see Table E for means and SDs per group and session).

Interestingly, this model further revealed quicker initiation time for threat vs. neutral displays (479 ± 140 ms vs. 396 ± 141 ms) in both poses and both sessions (β between -0.02 and -0.08, all p<0.001), which suggests that threat-related facial expressions significantly reduced the time needed to initiate an action. This threat effect decreased from session 1 to 2, as the significant interaction of threat and session in each pose group suggested (expansive: (β=0.02, 95% CI=0.00 – 0.03, t=2.24, p=0.025; contracted: β=0.03, 95% CI=0.01 – 0.04, t=3.75, p<0.001). Additionally, although the threat effect was highly significant in both poses, it was larger in the expansive pose group across both sessions (threat x pose interaction in session 1: β=0.03, 95% CI=0.01 – 0.04, t=3.98, p<0.001, and session 2: β=0.04, 95% CI=0.03 – 0.05, t=5.51, p<0.001). Yet, the fact that neither the main effect of pose nor the threat x session x pose interaction were significant (p>0.279) suggests that pose did not induce a change in threat detection. Means and confidence intervals of initiation time per emotion and side for each session and pose are depicted in Fig 4a.

Table B. Parameter estimates and model comparison for initiation time in angry and fearful trials.

Model of initiation time for angry and fearful trials: emotion x side

**Expansive pose and session 1 as baseline**				
Predictors	*β*	*CI*	*t*	*p*
(Intercept)	-1.01	-1.08 – -0.93	-26.28	**<0.001**
Emotion	0.00	-0.01 – 0.02	0.30	0.764
Side	0.00	-0.01 – 0.02	0.72	0.471
Session	-0.15	-0.17 – -0.14	-26.08	**<0.001**
Pose	0.02	-0.08 – 0.13	0.45	0.653
Emotion x Side	0.01	-0.01 – 0.03	0.78	0.436
Emotion x Session	-0.01	-0.03 – 0.01	-0.75	0.452
Side x Session	0.00	-0.01 – 0.02	0.30	0.765
Emotion x Pose	-0.00	-0.03 – 0.02	-0.17	0.868
Side x Pose	0.01	-0.00 – 0.03	1.57	0.117
Session x Pose	0.07	0.05 – 0.08	7.87	**<0.001**
Emotion x Side x Session	0.01	-0.02 – 0.04	0.73	0.465
Emotion x Side x Pose	-0.01	-0.04 – 0.02	-0.59	0.553
Emotion x Session x Pose	0.01	-0.02 – 0.04	0.52	0.605
Side x Session x Pose	-0.01	-0.03 – 0.02	-0.47	0.636

Emotion x Side x Session x Pose	-0.02	-0.06 – 0.03	-0.66	0.512
**Random Effects**				
σ^2	0.09			
$\tau_{00\ subject}$	0.06			
ICC	0.40			
$N_{subject}$	79			
Observations	41780			
Marginal R^2 / Conditional R^2	0.032 / 0.417			

Table C. Parameter estimates and model comparison for initiation time in all trials.

**Model of initiation time in all trials: threat vs. neutral**				
**Expansive pose and session 1 as baseline**				
Predictors	*β*	*CI*	*t*	*p*
(Intercept)	-0.97	-1.04 – -0.89	-25.86	**<0.001**
Threat	-0.08	-0.09 – -0.07	-15.70	**<0.001**
Session	-0.16	-0.17 – -0.15	-45.35	**<0.001**
Pose	0.02	-0.09 – 0.12	0.33	0.745
Threat x Session	0.02	0.00 – 0.03	2.24	**0.025**
Threat x Pose	0.03	0.01 – 0.04	3.98	**<0.001**
Session x Pose	0.06	0.05 – 0.07	11.55	**<0.001**
Threat x Session x Pose	0.01	-0.01 – 0.03	1.09	0.274
**Contracted pose and session 1 as baseline**				
Predictors	*β*	*CI*	*t*	*p*
(Intercept)	-0.95	-1.02 – -0.88	-25.07	**<0.001**
Threat	-0.05	-0.06 – -0.04	-9.85	**<0.001**
Session	-0.10	-0.11 – -0.10	-28.59	**<0.001**
Pose	-0.02	-0.12 – 0.09	-0.33	0.745
Threat x Session	0.03	0.01 – 0.04	3.75	**<0.001**
Threat x Pose	-0.03	-0.04 – -0.01	-3.98	**<0.001**
Session x Pose	-0.06	-0.07 – -0.05	-11.55	**<0.001**
Threat x Session x Pose	-0.01	-0.03 – 0.01	-1.09	0.274
**Expansive pose and session 2 as baseline**				
Predictors	*β*	*CI*	*t*	*p*
(Intercept)	-1.13	-1.20 – -1.05	-30.13	**<0.001**
Threat	-0.06	-0.07 – -0.05	-12.41	**<0.001**
Session	0.16	0.15 – 0.17	45.35	**<0.001**
Pose	0.08	-0.03 – 0.18	1.41	0.161
Threat x Session	-0.02	-0.03 – -0.00	-2.24	**0.025**
Threat x Pose	0.04	0.03 – 0.05	5.51	**<0.001**
Session x Pose	-0.06	-0.07 – -0.05	-11.55	**<0.001**
Threat x Session x Pose	-0.01	-0.03 – 0.01	-1.09	0.274
**Contracted pose and session 2 as baseline**				
Predictors	*β*	*CI*	*t*	*p*
(Intercept)	-1.05	-1.13 – -0.98	-27.76	**<0.001**
Threat	-0.02	-0.03 – -0.01	-4.56	**<0.001**
Session	0.10	0.10 – 0.11	28.59	**<0.001**
Pose	-0.08	-0.18 – 0.03	-1.41	0.161

Threat x Session	-0.03	-0.04 – -0.01	-3.75	**<0.001**
Threat x Pose	-0.04	-0.05 – -0.03	-5.51	**<0.001**
Session x Pose	0.06	0.05 – 0.07	11.55	**<0.001**
Threat x Session x Pose	0.01	-0.01 – 0.03	1.09	0.274
**Random Effects**				
σ^2	0.09			
$\tau_{00\ subject}$	0.06			
ICC	0.39			
Observations	62876			
Marginal R^2 / Conditional R^2	0.038 / 0.412			

The ANOVA on initiation time in angry and fearful trials (Table D), in contrast to the LMEM (Table B), indicated significantly slower initiation times for away than toward choices, i.e. a small effect of side. The significance of the side effect may differ between the ANOVA and the LMEM, as the first assesses the main effect of side across both groups and sessions, while the latter assesses simple effect of side in the expansive group in session 1. Setting other pose/session combinations as baseline (i.e. re-leveling pose and session) revealed no significant simple effect of side in the expansive group in session 2 (β=0.01, 95% CI= -0.00 – 0.02, t=1.13, p=0.257, in contrast to significant effects in the contracted group in both sessions (session 1: β=0.02, 95% CI=0.01 – 0.03, t=2.91, p=0.004; session 2: β=0.01, 95% CI=0.00 – 0.03, t=2.37, p=0.018). However, the effects in the contracted group were not significantly larger than those in the expansive group (side x pose in Table B, β=0.01, 95%CI= -0.00 – 0.0, t=1.57, p=0.117). Overall, these results indicate a small side effect.

Table D. ANOVAs initiation time in angry and fearful trials (assessing effects of emotion and side), and in all trials (comparing threat vs. neutral)

**ANOVAs initiation time**						
**In angry and fearful trials: emotion x side**						
	df		*F*	*p*	η^2_G	η^2_p
Pose	1	77	1.82	0.182	0.02	0.02
Emotion	1	77	0.70	0.404	0.00	0.01
Side	1	77	4.88	**0.030**	0.00	0.06
Session	1	77	45.38	**<0.001**	0.05	0.37
Pose x Emotion	1	77	2.32	0.132	0.00	0.03
Pose x Side	1	77	3.12	0.081	0.00	0.04
Pose x Session	1	77	2.23	0.139	0.00	0.03
Emotion x Side	1	77	0.20	0.652	0.00	0.00
Emotion x Session	1	77	0.11	0.740	0.00	0.00
Side x Session	1	77	0.64	0.425	0.00	0.01
Pose x Emotion x Side	1	77	2.24	0.139	0.00	0.03
Pose x Emotion x Session	1	77	0.01	0.938	0.00	0.00
Pose x Side x Session	1	77	0.01	0.946	0.00	0.00
Emotion x Side x Session	1	77	0.11	0.741	0.00	0.00
Pose x Emotion x Side x Session	1	77	0.13	0.725	0.00	0.00
**In all trials: threat vs. neutral**						
	df		*F*	*p*	η^2_G	η^2_p
Pose	1	77	1.05	0.308	0.01	0.01
Threat	1	77	83.34	**<0.001**	0.01	0.52
Session	1	77	62.97	**<0.001**	0.07	0.45

Pose x Threat	1	77	9.54	**0.003**	0.00	0.11
Pose x Session	1	77	2.08	0.153	0.00	0.03
Threat x Session	1	77	33.23	**<0.001**	0.00	0.30
Pose x Threat x Session	1	77	0.53	0.469	0.00	0.01

Table E. Means, SDs and CIs for initiation time in ms in threat vs. neutral trials. Within-subject 95% confidence intervals were calculated separately for each group according to the Cousineau-Morey method [22].

**Session**	**Group**	**Facial expression**	**n**	**Mean**	**SD**	**95% CI**
1 - Baseline	Expansive	Neutral	40	419	83	405-433
1 - Baseline	Expansive	Threatening	40	389	84	372-405
1 - Baseline	Contracted	Neutral	39	424	95	415-433
1 - Baseline	Contracted	Threatening	39	405	101	394-416
2 - Pose	Expansive	Neutral	40	358	78	342-373
2 - Pose	Expansive	Threatening	40	337	76	323-351
2 - Pose	Contracted	Neutral	39	378	92	369-387
2 - Pose	Contracted	Threatening	39	372	95	363-381

Movement duration

The LMEM on log-transformed movement duration (time from click to release on top of a chair) in angry and fearful trials (*log(movement duration) ~ (1|subject) + (1|stimulus pair) + emotion x side x session x pose*) converged including random intercepts for subject and stimuli, and was significantly better than the null model ($\chi^2(15)=49.069$, $p<0.001$, deviance (-2LL) reduced from -2156.1 to -2205.2). Given that neither emotion or side nor their interaction was significant (all β <-0.01, all p's > 0.386, see Table F, and H for the same analysis using ANOVA), we examined all other effects in the model including all trials.

This model (*log(movement duration) ~ (1|subject) + (1|stimulus pair) + threat x session x pose*) also performed significantly better than the null-model ($\chi^2(7)=103.51$, $p<0.001$, deviance (-2LL) reduced from -4253.8 to -4357.4, see Table G, and H for the same analysis using ANOVA). With the expansive group as baseline level for the treatment contrast for pose, this model revealed a significant effect of session (β=0.02, 95% CI=0.02 – 0.03, t=7.28, p<0.001), indicating slightly longer movement duration in the second session (session 1: 477 ± 159 ms, session 2: 483 ± 162 ms). The significant interaction of session and pose (β=-0.02, 95% CI=-0.03 – -0.01, t=-4.56, p<0.001) hinted that this session effect was not significant in the contracted group (β=0.00, 95% CI=-0.00 – 0.01, t=1.32 p=0.187, see Table I for means and SDs per group and session). A significant main effect of threat in both poses and sessions (all β=0.01, all p<0.014) indicated slower movement duration for threat than neutral displays (483 ± 163 ms vs. 477 ± 159 ms across both groups and sessions). In comparison to session and threat effects on initiation time, effects on movement duration were smaller and in the opposite direction: initiation time was quicker and movement duration slower for threat vs. neutral displays and in session 2. These opposite effects may be related to the fixed total time available to execute the movement; when clicking earlier, one has more time to move, and vice versa.

Table F. Parameter estimates and model comparison for movement duration in angry and neutral trials.

Model of movement duration for angry and fearful trials: emotion x side

**Expansive** pose **and session 1 as baseline**				
Predictors	*β*	*CI*	*t*	*p*
(Intercept)	-0.79	-0.88 – -0.71	-18.16	**<0.001**
Emotion	-0.01	-0.02 – 0.01	-0.87	0.386
Side	0.00	-0.01 – 0.01	0.41	0.684
Session	0.02	0.01 – 0.03	4.56	**<0.001**
Pose	-0.02	-0.14 – 0.10	-0.35	0.725
Emotion x Side	-0.00	-0.02 – 0.02	-0.05	0.963
Emotion x Session	0.01	-0.01 – 0.03	0.78	0.437
Side x Session	-0.00	-0.01 – 0.01	-0.06	0.950
Emotion x Pose	0.01	-0.01 – 0.03	1.00	0.318
Side x Pose	0.00	-0.01 – 0.01	0.35	0.728
Session x Pose	-0.02	-0.03 – -0.00	-2.69	**0.007**
Emotion x Side x Session	-0.00	-0.03 – 0.02	-0.34	0.735
Emotion x Side x Pose	-0.00	-0.03 – 0.02	-0.19	0.848
Emotion x Session x Pose	-0.02	-0.04 – 0.01	-1.39	0.166
Side x Session x Pose	-0.00	-0.02 – 0.02	-0.28	0.783
Emotion x Side x Session x Pose	0.02	-0.01 – 0.06	1.29	0.196
**Random Effects**				
σ^2	0.05			
$\tau_{00\ subject}$	0.08			
$\tau_{00\ stim\_pair}$	0.00			
ICC	0.58			
$N_{subject}$	79			
N_{stim_pair}	10			
Observations	41780			
Marginal R^2 / Conditional R^2	0.002 / 0.581			

Table G. Parameter estimates and model comparison for initiation time in all trials.

Model of movement duration in all trials: threat vs. neutral

**Expansive** pose **and session 1 as baseline**				
Predictors	*β*	*CI*	*t*	*p*
(Intercept)	-0.80	-0.88 – -0.71	-18.36	**<0.001**
Threat	0.01	0.01 – 0.02	3.25	**0.001**
Session	0.02	0.02 – 0.03	7.82	**<0.001**
Pose	-0.02	-0.14 – 0.10	-0.34	0.736
Threat x Session	-0.00	-0.01 – 0.01	-0.32	0.750
Threat x Pose	0.00	-0.01 – 0.01	0.10	0.924
Session x Pose	-0.02	-0.03 – -0.01	-4.56	**<0.001**
Threat x Session x Pose	-0.00	-0.02 – 0.01	-0.22	0.829
**Contracted** pose **and session 1 as baseline**				
Predictors	*β*	*CI*	*t*	*p*
(Intercept)	-0.82	-0.91 – -0.73	-18.60	**<0.001**
Threat	0.01	0.01 – 0.02	3.33	**0.001**
Session	0.00	-0.00 – 0.01	1.32	0.187

Pose	0.02	-0.10 – 0.14	0.34	0.736
Threat x Session	-0.00	-0.01 – 0.01	-0.62	0.537
Threat x Pose	-0.00	-0.01 – 0.01	-0.10	0.924
Session x Pose	0.02	0.01 – 0.03	4.56	**<0.001**
Threat x Session x Pose	0.00	-0.01 – 0.02	0.22	0.829
**Expansive** pose **and session 2 as baseline**				
Predictors	*β*	*CI*	*t*	*p*
(Intercept)	-0.78	-0.86 – -0.69	-17.86	**<0.001**
Threat	0.01	0.00 – 0.02	2.77	**0.006**
Session	-0.02	-0.03 – -0.02	-7.82	**<0.001**
Pose	-0.04	-0.16 – 0.08	-0.63	0.531
Threat x Session	0.00	-0.01 – 0.01	0.32	0.750
Threat x Pose	-0.00	-0.01 – 0.01	-0.21	0.834
Session x Pose	0.02	0.01 – 0.03	4.56	**<0.001**
Threat x Session x Pose	0.00	-0.01 – 0.02	0.22	0.829
**Contracted** pose **and session 2 as baseline**				
Predictors	*β*	*CI*	*t*	*p*
(Intercept)	-0.82	-0.90 – -0.73	-18.52	**<0.001**
Threat	0.01	0.00 – 0.02	2.46	**0.014**
Session	-0.00	-0.01 – 0.00	-1.32	0.187
Pose	0.04	-0.08 – 0.16	0.63	0.531
Threat x Session	0.00	-0.01 – 0.01	0.62	0.537
Threat x Pose	0.00	-0.01 – 0.01	0.21	0.834
Session x Pose	-0.02	-0.03 – -0.01	-4.56	**<0.001**
Threat x Session x Pose	-0.00	-0.02 – 0.01	-0.22	0.829
**Random Effects**				
σ^2	0.05			
$\tau_{00\ subject}$	0.08			
$\tau_{00\ stim\_pair}$	0.00			
ICC	0.58			
$N_{subject}$	79			
N_{stim_pair}	10			
Observations	62876			
Marginal R^2 / Conditional R^2	0.002 / 0.583			

Table H. ANOVAs movement duration in angry and fearful trials (assessing effects of emotion and side), and in all trials (comparing threat vs. neutral)

**ANOVAs movement duration**						
**In angry and fearful trials: emotion x side**						
	df		*F*	*p*	η^2_G	η^2_p
Pose	1	77	0.46	0.498	0.01	0.01
Emotion	1	77	0.00	0.995	0.00	0.00
Side	1	77	0.87	0.354	0.00	0.01
Session	1	77	0.63	0.432	0.00	0.01
Pose x Emotion	1	77	1.21	0.276	0.00	0.02
Pose x Side	1	77	0.98	0.325	0.00	0.01
Pose x Session	1	77	1.44	0.234	0.00	0.02
Emotion x Side	1	77	0.06	0.811	0.00	0.00
Emotion x Session	1	77	0.35	0.554	0.00	0.01

Side x Session	1	77	1.55	0.218	0.00	0.02
Pose x Emotion x Side	1	77	1.88	0.174	0.00	0.02
Pose x Emotion x Session	1	77	2.62	0.110	0.00	0.03
Pose x Side x Session	1	77	0.48	0.491	0.00	0.01
Emotion x Side x Session	1	77	0.63	0.429	0.00	0.01
Pose x Emotion x Side x Session	1	77	0.77	0.383	0.00	0.01
**In all trials: threat vs. neutral**						
	df		*F*	*p*	η^2_G	η^2_p
Pose	1	77	0.48	0.492	0.01	0.01
Threat	1	77	48.59	**<0.001**	0.00	0.39
Session	1	77	0.86	0.358	0.00	0.01
Pose x Threat	1	77	0.00	0.985	0.00	0.00
Pose x Session	1	77	1.24	0.270	0.00	0.02
Threat x Session	1	77	0.75	0.389	0.00	0.01
Pose x Threat x Session	1	77	0.61	0.437	0.00	0.01

Table I. Means, SDs and CIs for movement duration in ms in threat vs. neutral trials. Within-subject 95% confidence intervals were calculated separately for each group according to the Cousineau-Morey method [22].

**Session**	**Group**	**Facial expression**	**n**	**Mean**	**SD**	**95% CI**
Expansive	1 - Baseline	Neutral	40	476	125	462-489
Expansive	1 - Baseline	Threatening	40	482	127	468-495
Contracted	1 - Baseline	Neutral	39	464	112	451-477
Contracted	1 - Baseline	Threatening	39	471	115	457-485
Expansive	2 - Pose	Neutral	40	489	137	477-502
Expansive	2 - Pose	Threatening	40	495	139	481-509
Contracted	2 - Pose	Neutral	39	464	110	450-478
Contracted	2 - Pose	Threatening	39	469	113	455-482

Supplementary References

1. Vilarem E, Armony JL, Grèzes J. Action opportunities modulate attention allocation under social threat. Emotion. 2019 [cited 23 May 2019]. doi:10.1037/emo0000598
2. Chen M, Bargh JA. Consequences of Automatic Evaluation: Immediate Behavioral Predispositions to Approach or Avoid the Stimulus. Personality and Social Psychology Bulletin. 1999;25: 215–224. doi:10.1177/0146167299025002007
3. Solarz AK. Latency of instrumental responses as a function of compatibility with the meaning of eliciting verbal signs. Journal of Experimental Psychology. 1960;59: 239–245. doi:10.1037/h0047274
4. Phaf RH, Mohr SE, Rotteveel M, Wicherts JM. Approach, avoidance, and affect: a meta-analysis of approach-avoidance tendencies in manual reaction time tasks. Front Psychol. 2014;5. doi:10.3389/fpsyg.2014.00378
5. De Houwer J, Crombez G, Baeyens F, Hermans D. On the generality of the affective Simon effect. Cognition & Emotion. 2001;15: 189–206. doi:10.1080/02699930125883

6. Marsh AA, Adams RB, Kleck RE. Why do fear and anger look the way they do? Form and social function in facial expressions. Pers Soc Psychol Bull. 2005;31: 73–86. doi:10.1177/0146167204271306

7. Seibt B, Neumann R, Nussinson R, Strack F. Movement direction or change in distance? Self- and object-related approach–avoidance motions. J Exp Soc Psychol. 2008;44: 713–720. doi:10.1016/j.jesp.2007.04.013

8. Kozlik J, Neumann R, Lozo L. Contrasting motivational orientation and evaluative coding accounts: on the need to differentiate the effectors of approach/avoidance responses. Frontiers in Psychology. 2015;6. doi:10.3389/fpsyg.2015.00563

9. Laham SM, Kashima Y, Dix J, Wheeler M. A meta-analysis of the facilitation of arm flexion and extension movements as a function of stimulus valence. Cognition and Emotion. 2015;29: 1069–1090. doi:10.1080/02699931.2014.968096

10. Bossuyt E, Moors A, De Houwer J. On angry approach and fearful avoidance: The goal-dependent nature of emotional approach and avoidance tendencies. Journal of Experimental Social Psychology. 2014;50: 118–124. doi:10.1016/j.jesp.2013.09.009

11. Krieglmeyer R, De Houwer J, Deutsch R. On the Nature of Automatically Triggered Approach–Avoidance Behavior. Emotion Review. 2013;5: 280–284. doi:10.1177/1754073913477501

12. Paulus A, Wentura D. It depends: Approach and avoidance reactions to emotional expressions are influenced by the contrast emotions presented in the task. Journal of Experimental Psychology: Human Perception and Performance. 2016;42: 197–212. doi:10.1037/xhp0000130

13. Wilkowski BM, Meier BP. Bring it on: Angry facial expressions potentiate approach-motivated motor behavior. Journal of Personality and Social Psychology. 2010;98: 201–210. doi:10.1037/a0017992

14. Spielberger CD, Gorush RL, Lushene R, Vagg P, Jacobs GA. Manual for the State-Trait Anxiety Inventory. Palo Alto, CA: Consulting Psychologists Press; 1983.

15. Caci H, Deschaux O, Baylé FJ. Psychometric properties of the French versions of the BIS/BAS scales and the SPSRQ. Personality and Individual Differences. 2007;42: 987–998. doi:10.1016/j.paid.2006.09.008

16. Vallières ÉF, Vallerand RobertJ. Échelle d'estime de soi. International Journal of Psychology. 1990;25: 305–316.

17. Jacobs I, Scholl W. IAL-K: Entwicklung einer Kurzform der Interpersonalen Adjektivliste. Diagnostica. 2016;62: 227–241. doi:10.1026/0012-1924/a000156

18. Barr DJ. Random effects structure for testing interactions in linear mixed-effects models. Front Psychol. 2013;4. doi:10.3389/fpsyg.2013.00328

19. Kuznetsova A, Brockhoff PB, Christensen RHB. Lmertest package: Tests in linear mixed effects models. Journal of Statistical Software. 2017;82. doi:10.18637/jss.v082.i13

20. Lakens D. Equivalence tests: A practical primer for t-tests, correlations, and meta-analyses. PsyArXiv. 2016 [cited 19 Mar 2018]. doi:10.1177/1948550617697177

21. DeBruine LM, Barr DJ. Understanding Mixed-Effects Models Through Data Simulation. Advances in Methods and Practices in Psychological Science. 2021;4: 2515245920965119. doi:10.1177/2515245920965119

22. Baguley T. Calculating and graphing within-subject confidence intervals for ANOVA. Behavior Research Methods. 2012;44: 158–175. doi:10.3758/s13428-011-0123-7
