# Supplementary material for: Power pose effects on approach and avoidance decisions in response to social threat
Source: PLoS One. 2023 Aug 18;18(8):e0286904. doi: 10.1371/journal.pone.0286904 (PMC10437884; doi:10.1371/journal.pone.0286904)
Supplement: S1 File — (PDF) [file pone.0286904.s002.pdf]

Behavioural Science Institute

Radboud University Nijmegen
Behavioural Science Institute

Faculty of Social Sciences

Dr. Gijsbert Bijlstra
Thomas van Aquinostraat 4
6525 GD NIJMEGEN
P.O. Box 9104
The Netherlands

Telephone +31 24 36 10081

www.ru.nl/fsw

Our reference	Your reference	Telephone	Date
		+31 24 36 10081	July 5, 2021
Subject: Permission to print RaFD stimulus example		E-mail<br>info@rafd.nl	

To whom it may concern,

We, the undersigned, hereby grant the permission to publish images of stimuli created by Emma Vilarem using images from our Radboud Faces Database. These images may be published in all formats, i.e. print and digital formats under an Open Access Creative Commons license (CC-BY).

Yours faithfully,

Dr. Gijsbert Bijlstra
