# Supplementary material for: Power pose effects on approach and avoidance decisions in response to social threat
Source: PLoS One. 2023 Aug 18;18(8):e0286904. doi: 10.1371/journal.pone.0286904 (PMC10437884; doi:10.1371/journal.pone.0286904)
Supplement: Attachment — Submitted filename: Response_letter_reviewers_r2.pdf [file pone.0286904.s004.pdf]

Revision 2 – PONE-D-21-19478R1

May 3rd 2023

Rebuttal letter: Responses to reviewers – revision 2

Power pose effects on approach and avoidance decisions in response to social threat

Journal Requirements:

Please review your reference list to ensure that it is complete and correct. If you have cited papers that have been retracted, please include the rationale for doing so in the manuscript text, or remove these references and replace them with relevant current references. Any changes to the reference list should be mentioned in the rebuttal letter that accompanies your revised manuscript. If you need to cite a retracted article, indicate the article's retracted status in the References list and also include a citation and full reference for the retraction notice.

We have checked our reference list. It includes no retracted papers (based on labels of retracted papers in Zotero, which are based on Retraction Watch). We also searched http://retractiondatabase.org for important terms in our publication, and found no retracted studies. Search terms: power pose, social power, anger, fear, power, facial expression, avoidance, dominance.

The current revision involves 3 changes to the reference list:

1) These 5 references were removed:

- LeDoux J, Daw ND. Surviving threats: neural circuit and computational implications of a new taxonomy of defensive behaviour. Nat Rev Neurosci. 2018;19: 269–282. doi:10.1038/nrn.2018.22
- Pessiglione M, Schmidt L, Draganski B, Kalisch R, Lau H, Dolan RJ, et al. How the Brain Translates Money into Force: A Neuroimaging Study of Subliminal Motivation. Science. 2007;316: 904–906. doi:10.1126/science.1140459
- Wimmer GE, Shohamy D. Preference by Association: How Memory Mechanisms in the Hippocampus Bias Decisions. Science. 2012;338: 270–273. doi:10.1126/science.1223252
- Balleine BW, Dickinson A. Goal-directed instrumental action: contingency and incentive learning and their cortical substrates. Neuropharmacology. 1998;37: 407–419. doi:10.1016/S0028-3908(98)00033-1
- Cisek P. Cortical mechanisms of action selection: the affordance competition hypothesis. Philosophical Transactions of the Royal Society of London B: Biological Sciences. 2007;362: 1585–1599. doi:10.1098/rstb.2007.2054
- Rangel A, Camerer C, Montague PR. A framework for studying the neurobiology of value-based decision making. Nat Rev Neurosci. 2008;9: 545–556. doi:10.1038/nrn2357

2) This reference was added to the reference list:
Körner R, Röseler L, Schütz A. Commentary on Elkjær et al.'s (2020) Meta-Analysis on Expansive Versus Contractive Nonverbal Displays. Perspect Psychol Sci. 2022;17: 305–307. doi:10.1177/1745691620984474

3) For the reference Elkjær et al.'s (2022), the year of publication was changed from the date it was published in a journal Volume (2022) to the date of its online publication (2020), to make clear that the Commentary mentioned under point 2) above refers to Elkjær et al. (2020), and not another publication:

Revision 2 – PONE-D-21-19478R1

Elkjær E, Mikkelsen MB, Michalak J, Mennin DS, O'Toole MS. Expansive and Contractive Postures and Movement: A Systematic Review and Meta-Analysis of the Effect of Motor Displays on Affective and Behavioral Responses: Perspectives on Psychological Science. 2020;17: 276–304. doi:10.1177/1745691620919358

Please note that reference numbers between the final unmarked manuscript and the marked-up manuscript with tracked changes differ. The marked-up version still contains some references that were cut in the final version. Please refer to the reference list and numbers in the final version for all steps related to publication.

Review Comments to the Author

A note regarding editor and reviewers:

Reference numbers and page numbers below refer to the marked-up manuscript with tracked changes. Reference numbers in the final unmarked manuscript differ, because marked-up version still contains the references that were cut.

Reviewer #1:

The authors took great care in responding to the raised critical points. I really like the present version of the manuscript. The authors now report the different aspects of power-posing research, the problems of replicability and the gap their research fills in much more detail. They are also more transparent with regard to the exploratory aspects of their study and the slight underpowering of their research. Importantly, they also provide more background about the possibly underlying processes which allows readers to compare it to other power-posing research (i.e., perhaps power-posing effects are based on different mechanisms depending on the paradigm). I find it thus very important that the authors outline the differences between their approach-avoidance task and other paradigms. Their action decision is one a more conceptual level (i.e., you have to know that sitting on that chair reduces/increases distance) while a constant body posture is taken, whereas in other's the arm movement is the DV.

As a small point: I think there is no need to expand that much on Rohr et al. 2015 and related articles. :-) (I think you can delete or shorten to one or two sentences). I really was just curious on how participants processed the expressions. So, it seems that it was attentional unawareness (i.e., like in the inattentional blindness Gorilla movie) for most participants.

Dear Dr. Rohr,

Thank you very much for reviewing our manuscript a second time, and for your constructive and positive feedbacks. Regarding the last small point: we shortened the description of how participants processed the expressions in the manuscript, and have inserted this part of the manuscript below. We also thank you for suggesting the term attentional unawareness, which we may use in the future to describe our findings.

Best,
Hannah Metzler

The shortened section now reads (page 6):

Mennella et al. [54] and Grèzes et al. [53] further revealed that angry and to a lesser extent fearful displays, increased the expected value of the action leading to avoidance. In other words, sitting far from a threatening individual was a highly motivational (desirable) outcome per se, even in the context of a laboratory task. ~~Consistent with the proposal that flexible decision processes can include both explicit and implicit forecasting of action outcomes [54], these results provide evidence for an emotion-based process of value attribution that can influence action (approach/avoidance) selection very rapidly, and possibly outside consciousness [56,57].~~

Research further suggests that~~, during the process of decision-making, the brain specifies and assigns value to the potential actions available in the world using relevant information from both external (for instance, threat displays) and internal (i.e., the motivational, emotional, and cognitive state) contexts of the decision-maker [58,60]. A~~ action possibilities are constrained by the individual's action capabilities [61], which can be rapidly recalibrated as a function of external [e.g., 62] or internal factors [63,64].

Reviewer #2:

A note regarding editor and reviewers:

Reference numbers and page numbers below refer to the marked-up manuscript with tracked changes. Reference numbers in the final unmarked manuscript differ, because marked-up version still contains the references that were cut.

Thank you for inviting me to re-review the manuscript titled, "Power pose effects on approach and avoidance decisions in response to social threat". I read the response letter and the revised manuscript. Overall, I feel that the authors have addressed my concerns almost completely.

Dear Dr. Körner,

Thank you for reviewing our manuscript a second time, your comments helped improve our manuscript once more.

However, I still have a few minor points:

1) In response to a large point by Reviewer 1, the authors wrote, "The present study was conducted during the ongoing replication debate (in 2016), and needs to be interpreted in this context." This sounds somewhat like, "The study is not methodological up-to-date but it was several years ago and this is why it should be published because it fulfills publishing criteria some years ago". I think this is problematic because the study needs now – in 2023 – to convince the scientific community. The authors should remove the sentence and refer to limitations in the Discussion. Perhaps it might also help to name the study a "pilot study" or "pilot experiment".

We have removed the sentence, and rephrased the limitation section in the discussion. This involved moving the sentences marked in blue from lines 498-503 to the limitations section (line 517-525), and instead cutting and restructuring some other sentences.

Revision 2 – PONE-D-21-19478R1

The limitation section now reads (page 22, line 516): *"Despite these strengths, this study is not without limitations. First, within-subject designs were suggested to increase the risk of demand effects [15]. In the past, the majority of studies on postural feedback effects used explicit self-reports about behaviors* [97–100]*, which appear to be susceptible to demand effects. Indeed, the pose effect on explicit feelings of power was found to be larger in subjects familiar with "power posing", in a meta-analysis of six pre-registered and highly-powered studies [30], as well as in a similarly large pre-registered study [29]. In the present study, however, only 2 participants suspected a link between the pose manipulation and the approach-avoidance task even after extensive debriefing (see S1 Appendix), indicating that the influence of poses on action decisions in response to task-irrelevant stimuli may reduce such demand effects. ~~Investigating the influence of poses on action decisions in response to task-irrelevant stimuli such as in the present study reduces such demand effects.~~*

~~we want to mention that the present study was conducted during the ongoing replication debate (in 2016), and might have been designed differently if conducted more recently. For instance, However, within-subject designs may also make demand effects more likely [15]. Since we measured actual behavior rather than self-reports, and only 2 participants suspected a link between the pose manipulation and the approach-avoidance task even after extensive debriefing (see S1 Appendix), demand effects are less likely to explain our results.~~ Second, we only included male participants in the present study. This decision was due to the study being part of a larger project on power poses [70], which began before it was clear that effects on cortisol and testosterone did not replicate. We initially suspected hormonal mechanisms to underlie the poses' effects on social behavior, and biological differences in the production of testosterone and the menstrual cycle require separate hormonal analyses by gender [104]. Therefore, most studies in the project focused only on men, whereas those that included women observed gender effects in face perception [70, Chapter 4]. Wanting to achieve robust results and maximize sample size per group in the context of the replication debate, within our feasibility constraints, we therefore included only men. As Körner et al.'s [15] meta-analysis revealed that power pose effects do not depend on gender, it seems possible that our findings may generalize to women."

2) I am not convinced with the authors' response to my comment 7. If the authors still decide to keep the Elkjaer et al. (2022) meta-analysis in the manuscript they should accompany the reference with the criticism (https://doi.org/10.1177/1745691620984474).

Further, it seemed that there is a misunderstanding in reading the commentary: Not the lack of open science practices in empirical power posing studies is criticized but the lack of open science practices in the Elkjaer et al. (2022) meta-analysis is criticized. The authors did not share their data and analysis code (also not on request), which is why we cannot fully trust such a recent paper in this controversial field, in which preregistration and open science practices are very important to regain trust. Moreover, as the Elkjaer et al. (2022) meta-analysis includes not even 50% of the studies analyzed in the more recent meta-analysis (Körner et al., 2022), it seems more than questionable why contracted body positions might have an effect. Thus, I strongly recommend to (1) refer to the meta-analysis only together with the

critical commentary because otherwise readers might be misled, and (2) do not claim at any place in the manuscript that other meta-analyses suggest the effect in power posing studies might be driven by the contractive body position group (we still don't know this but your own study might add to the open question).

We are sorry that our response was not clear. We fully agree that open science practices are important to regain trust in research on power poses, and understood that this was about Elkjaer et al. (2020) itself. Regarding your recommendations, in the current revision, we made sure that we accompany the reference to Elkjaer et al. (2020) with your critical commentary (https://doi.org/10.1177/1745691620984474), and have removed one mention of Elkjaer et al. (2020) in the introduction. Our earlier revision cited your meta-analysis instead of the commentary, this was an unintentional mistake for which we apologize. We also now use the year of its online publication (2020), to make clear that your commentary that mentions the year 2020 in its title refers to this publication. However, we think that not mentioning Elkjaer et al. (2020)'s findings at all is unjustified, notably in the discussion. We find the inclusion criteria applied in Elkjaer et al., (2020) reasonable for the specified purpose of aggregating only effects of body-feedback, only effects of whole body manipulations, and only behavioral or affective outcomes. The meta-analysis thus focuses on a smaller, but consistent set of studies, which are quite similar to our current study, and therefore important to mention once.

Here our edits in the manuscript with added text and deleted text marked:

- Introduction (page 3): "*Two recent meta-analyses ~~summarizing all studies fulfilling certain criteria~~ provide evidence for a statistical difference between expansive and contracted poses for self-reported feelings and overt behavioral outcomes, but not for hormones, after correcting for publication bias (n=96 reports [15], n=48 reports [27, but see 28]). Both highlighted a lack of studies that allow definitive conclusions about whether expansive or contracted power poses drive the effects. ~~Preliminary findings based on only a few studies hint that only contracted poses may induce effects [27].~~*"

- Discussion (page 19, line 417): "*~~The fact that e~~Expansive poses did not significantly decrease avoidance of angry individuals, in contrast to our predictions~~,~~. While our result appears to be consistent with ~~is consistent with preliminary evidence from~~ a recent meta-analysis ~~of the power posing literature which hints~~suggesting that only contracted poses may change behavior in comparison to neutral poses [27], the issues raised about this meta-analysis [28], and the small number of studies including neutral conditions prevent any definitive conclusion at this point.*"

  In its final version, this paragraph reads as follows: "*Expansive poses did not significantly decrease avoidance of angry individuals, in contrast to our predictions. While our result appears to be consistent with a recent meta-analysis suggesting that only contracted poses may change behavior in comparison to neutral poses [27], the issues raised about this meta-analysis [28], and the small number of studies including neutral conditions prevent any definitive conclusion at this point.*"

Revision 2 – PONE-D-21-19478R1

3) I am not a native English speaker but I feel that the manuscript needs to be proofread.

Signed, Robert Körner

We have proofread the manuscript, and run a grammar correction and language improvement tool over it. All language improvement changes are marked in the manuscript with tracked changes.

---

7. PLOS authors have the option to publish the peer review history of their article (what does this mean?). If published, this will include your full peer review and any attached files.

If you choose "no", your identity will remain anonymous but your review may still be made public.

Do you want your identity to be public for this peer review? For information about this choice, including consent withdrawal, please see our Privacy Policy.

Reviewer #1: **Yes:** Dr. Michaela Rohr

Reviewer #2: No
